# NLRP3 inflammasome activation by particles and fibers: implications for human hazard assessment

**DOI:** 10.1007/s00204-026-04477-x

**Published:** 2026-06-13

**Authors:** Mathias Busch, Gerrit Bredeck, Hans Bouwmeester, Roel P. F. Schins

**Affiliations:** 1 Division of Toxicology, Wageningen University and Research, Droevendaalsesteeg 4, 6708 PB Wageningen, The Netherlands; 2 Centro de Investigaciones Biológicas Margarita Salas (CIB-CSIC), Calle de Ramiro de Maeztu, 9, Moncloa - Aravaca, 28040 Madrid, Spain; 3 Department of Pharmacology and Toxicology, NUTRIM Institute of Nutrition and Translational Research in Metabolism, Maastricht University, Minderbroedersberg 4-6, 6211 LK Maastricht, The Netherlands; 4 IUF – Leibniz Research Institute for Environmental Medicine, Auf’m Hennekamp 50, 40225 Düsseldorf, Germany

**Keywords:** Inflammation, Asbestos, Microplastics, Silica, Titanium dioxide, Desert dust

## Abstract

The NOD-, LRR- and pyrin domain-containing protein 3 (NLRP3) inflammasome has been shown to be involved in the manifestation of adverse effects following exposure to occupational, dietary and environmental particulate matter. In this review, we summarize knowledge on NLRP3 inflammasome activation by particles and fibers in vitro and in vivo, outline dominant activation mechanisms and discuss the link to human diseases. While the association between NLRP3 activation and development of diseases is well established for occupational exposure to mineral dusts like asbestos and quartz, the health effects of low-level exposure to environmental particulate pollutants, such as micro- and nanoplastics, desert dust or novel nanocomposites remain to be unraveled. Based on the combined literature of well-studied and emerging particles, we emphasize the relevance of testing for NLRP3 activation in the hazard assessment of novel particulate materials.

## Introduction

The NOD-, LRR- and pyrin domain-containing protein 3 (NLRP3) inflammasome is a multiprotein complex located in the cytosol (He et al. 2016). It is a component of the innate immune system, primarily present in immune cells, that has been shown to play a significant role in the toxicological effects of mineral dusts and fibers, eventually leading to diseases in humans upon chronic activation (Sayan and Mossman 2016). The discovery of the NLRP3 inflammasome represents an important milestone in understanding inflammation, innate immunity and the potential adverse human health consequences upon particle exposure.

The concept of inflammasomes was first introduced by Jürg Tschopp and colleagues (Martinon et al. 2002), describing multiprotein complexes capable of activating caspase-1, leading to cleavage of pro-interleukin(IL)-1β (Martinon et al. 2002). The authors subsequently identified inflammasomes as a platform comprised of members of the NOD-like receptor (NLR) family. Among these receptors, NLRP3 specifically gained attention due to its responsiveness towards a myriad of stimuli, including pathogens, particles and environmental pollutants (Anand et al. 2011; Ma and Lim 2024; Mou et al. 2023). The main consequence of NLRP3 activation is the release of the pro-inflammatory cytokines IL-1β and IL-18, important early mediators of several immunological reactions (He et al. 2016). In general, the activation of NLRP3 requires two steps: (i) priming of the inflammasome and (ii) subsequent activation of the inflammasome (Wagatsuma and Nakase 2020).

The priming step is necessary to induce the production of the inactive NLRP3 protein, as well as pro-IL-1β and, depending on the cell type, pro-IL-18, the immature forms of IL-1β and IL-18. Priming is facilitated through Nuclear factor-kappa light chain enhancer of activated B cells (NF-κB), a cytosolic transcription factor. NF-κB can be activated by danger-associated molecular patterns (DAMPs, e.g. extracellular adenosine triphosphate (ATP)) or pathogen-associated molecular patterns (PAMPs) (e.g. lipopolysaccharide (LPS)) binding to pattern recognition receptors (PRRs), such as the Toll-like receptor 4 (TLR4) (McKee and Coll 2020). Cytokines, such as tumor necrosis factor (TNF) and IL-1β itself are also able to prime the NRLP3 inflammasome via NF-κB activation (McGeough et al. 2017).

The activation step of the NLRP3 inflammasome can be induced by a variety of different stimuli. DAMPs and PAMPs, such as extracellular ATP or the bacterial toxin nigericin, mineral dusts such as crystalline silica (SiO_2_) and asbestos (Sayan and Mossman 2016) but also nanoparticles such as nanosize titanium dioxide (n-TiO_2_) or nanosize amorphous silica (n-SiO_2_), can trigger intracellular events that lead to NLRP3 activation (Baron et al. 2015). These events include rupture of lysosomes, leading to the release of lysosomal Cathepsin B and efflux of potassium (K^+^) ions, mitochondrial damage leading to the production of reactive oxygen species (ROS), or endoplasmatic reticulum stress mediated by thioredoxin interacting protein (TXNIP) dissociating from thioredoxin (TRX) (Choi and Park 2023). In the event of activation, NLRP3 units oligomerize, and the pyrin domain recruits the apoptosis-associated speck-like protein (ASC), which in turn recruits pro-caspase-1, the inactive form of caspase-1. After recruitment, pro-caspase-1 autocatalytically cleaves itself, resulting in the active form of caspase-1. Active caspase-1 is capable of cleaving its substrates, pro-IL-1β and pro-IL-18, causing the release of mature IL-1β and IL-18 into the extracellular environment (Lopez-Castejon and Brough 2011), e.g. via exosomes, microvesicles or terminal release after undergoing pyroptosis (Bergsbaken et al. 2009; MacKenzie et al. 2001; Qu et al. 2007).

Pyroptosis is a specific type of cell death mediated via the cleavage of Gasdermin D (GSDMD) by caspase-1, which then forms pores in the cell membrane, ultimately leading to lysis of the cell (Vince and Silke 2016). The released pro-inflammatory cytokines IL-1β and IL-18 induce subsequent auto- and paracrine cascades that promote further inflammation of the surrounding tissue, such as the NF-κB pathway and mitogen-activated protein kinase (MAPK) cascade. This leads to the release of additional pro-inflammatory cytokines (IL-6 and TNF-α) and chemokines (IL-8) to attract immune cells, or the activation of cyclooxygenase 2 (COX2) and subsequent prostaglandin production (Collart et al. 1990; Liu et al. 2015, 2014; Newton et al. 1997). Prostaglandins are involved in regulating the classic signs of tissue inflammation, including vasodilation, pain and fever (Ricciotti and FitzGerald 2011).

## NLRP3 activation by occupational dusts, fibers and particulate matter

In numerous in vivo and in vitro studies, it has been shown that adverse reactions following particle exposure, including the development of immunological diseases, are involving the activation of the NLRP3 inflammasome pathway. The activation of the NLRP3 inflammasome has been extensively studied for some prominent particles, such as the “Big 3” in occupational inhalation toxicology: crystalline silica dusts (quartz and cristobalite), asbestos fibers and coal mine dust (Cassel et al. 2008; Dostert et al. 2008; Peeters et al. 2014; Zhang et al. 2025). For these particle and fiber types, activation of NLRP3 and a link to lung diseases in humans have been well established.

In several different in vivo and in vitro models, crystalline silica particles have been shown to destabilize lysosomes after cellular uptake, causing the release of lysosomal components into the cytosol that, combined with generating ROS on the surface, leads to NLRP3 activation and ultimately IL-1β release (Bredeck et al. 2023a; Busch et al. 2022b; Dostert et al. 2008; Hornung et al. 2008; Kolling et al. 2020; Peeters et al. 2014, 2013; Zhou et al. 2023). Due to small size, quartz dust is able to penetrate deep into the lung. Combined with insolubility and limited clearance, this leads to continuous inflammation in the lung, eventually leading to silicosis, a type of fibrotic lung disease and one of the most important occupational diseases worldwide (Leung et al. 2012).

Asbestos fibers have been shown to cause frustrated phagocytosis in various macrophage models, leading to NADPH-dependent generation of ROS, Cathepsin B release, NLRP3 and Caspase-1 activation and finally, secretion of IL-1β (Dostert et al. 2008; Palomäki et al. 2011; Thompson et al. 2014). Frustrated phagocytosis describes the failure of an immune cell to completely engulf long, rigid fibers, leading to mechanical damage to the phagosome and cell membrane (Padmore et al. 2017). Similar to silicosis, asbestosis is an important occupational lung disease caused by excessive inhalation of asbestos fibers, leading to interstitial fibrosis of the lung, a well described precursor of lung cancer (Mossman and Churg 1998).

Coal mine dust is capable of generating intracellular ROS in the lung, causing an activation of NLRP3, leading to Caspase-1 activation and IL-1β release (Zhang et al. 2022a). Potentially, this causes coal workers pneumoconiosis, also known as “black lung disease”, which is characterized by severe fibrosis in lung tissue and impaired respiratory function (Vanhee et al.).

In the case of TiO_2_, an important nanomaterial in medical, paint, cosmetics and food industries, every crucial step in the NLRP3 cascade, i.e. intracellular accumulation of particles, generation of ROS, upregulation of NLRP3-associated genes, cleavage of Caspase-1, as well as cleavage and secretion of IL-1β, has been observed after exposure of various in vitro and in vivo models to TiO_2_ (Abbasi-Oshaghi et al. 2019; Busch et al. 2022a; Duan et al. 2023; Kim et al. 2017; Kolling et al. 2020; Morishige et al. 2010b; Ruiz et al. 2017; Yazdi et al. 2010). Of note, there are different crystal forms of TiO_2_, such as rutile, anatase or brookite (Diebold 2003), which influence the NLRP3 activating potential (Kolling et al. 2020). Food grade TiO_2_ (E171) is a mixture of micro- and nanoparticles widely used as a food additive. While E171 is internalized by cells and can generate ROS (Proquin et al. 2016; Rodríguez-Ibarra et al. 2022), there is no clear evidence of NLRP3 activation in literature yet, apart from studies using nano TiO_2_ only.

Monosodium urate (MSU) crystals, a popular positive control for studying particle-induced NLRP3 activation, have been shown to be internalized by cells, rupture the lysosome, cause mitochondrial damage, ROS and K^+^ efflux, which all lead to activation of NLRP3 with subsequent IL-1β and IL-18 release (Kim et al. 2020; Martinon et al. 2006; Pan et al. 2021; Pétrilli et al. 2007; Scott et al. 2006; Zheng et al. 2015). MSU crystals are endogenously formed particles as a consequence of renal and intestinal urate underexcretion, and are responsible for joint inflammation in gout (Narang and Dalbeth 2020). Other endogenous particles capable of activating NLRP3 are calcium pyrophosphate (CPP) crystals involved in calcium pyrophosphate deposition disease, also known as pseudogout (Pascart et al. 2024).

For a wide panel of other particle types in occupational settings, such as carbon black, environmental particles such as diesel exhaust or fly ash, and engineered nanomaterials, including synthetic amorphous silica, carbon nanotubes, silver nanoparticles, alum or various metal oxides, NLRP3 activating potential has been demonstrated in a vast number of in vitro and vivo studies (Table 1). Ambient particulate matter (PM2.5), i.e. air contaminants able to penetrate deep into the lung due to their small size of ≤ 2.5 µm, represents a mixture of anthropogenic and non-anthropogenic solid particles and lipid droplets. This mixture has also been shown to trigger lung inflammation via the NLRP3 pathway (Cao et al. 2022; Jia et al. 2021; Li et al. 2020a; Niu et al. 2021; Shen et al. 2019; Wang et al. 2020; Zheng et al. 2018). On the other hand, newly identified potential hazards, such as environmental dust from the Saharan desert, have been less extensively studied. These particles showed Caspase-1 and NLRP3 inflammasome-dependent IL-1β secretion in macrophage-like cells (Bredeck et al. 2023a, 2023b). Desert dust exposure has been epidemiologically associated with chronic respiratory and cardiovascular morbidity, and prolonged exposure with dust-related pneumoconiosis (Bell et al. 2008; Norboo et al. 1991; Thalib and Al-Taiar 2012). Volcanic ash represents another non-anthropogenic dust mixture that was shown to activate the NLRP3 inflammasome (Damby et al. 2018). Its inflammasome-activating properties were found to be linked to its large proportion of cristobalite, a distinct type of crystalline SiO_2_ formed at high temperatures.

**Table 1:** NLRP3 inflammasome activation by particles

Particle type	Used models	Observed events	References
Crystalline silica (SiO_2_) as quartz or cristobalite	Female Wistar rats (intratracheal instillation), C57BL/6 mice (WT, *Nalp3*^*−/−*^, *Asc*^*−/−*^, *Nlrp3-*CHCl reporter), THP-1 cells (WT, *NLRP3*^*−/−*^,*CASP1*^*−/−*^), BEAS-2B cells, primary human bronchial epithelial cells, human PBMCs, murine bone marrow-derived macrophages (WT, *Nlrp3*^*−/−*^, *Nlrp3-*CHCl reporter), murine tissue-derived lung organoids, murine peritoneal macrophages (WT, *Nlrp3*^*−/−*^), human alveolar macrophages, murine bone marrow-derived dendritic cells (WT, *Nlrp3*^*−/−*^)	Cellular uptake via phagocytosis Lysosomal destabilization Production of and dependence on ROS Cathepsin B dependence K^+^ dependence Increased *NLRP3* gene expression Involvement of TXNIP NLRP3 activation Involvement of ASC Caspase-1 activation IL-1β cleavage IL-1β secretion	Bredeck et al. (2023a), Busch et al. (2022b), Cassel et al. (2008), Dostert et al. (2008), Hornung et al. (2008), Kolling et al. (2020), Lam et al. (2025), Peeters et al. (2014), Peeters et al. (2013), Winter et al. (2011), Zhou et al. (2023)
Asbestos fibers	Human macrophages, THP-1 cells, murine bone marrow-derived macrophages, HPM3 cells, C57BL/6 mice (WT, *Nlrp3*^*−/−*^, *Casp1*^*−/−*^, *Asc*^*−/−*^), human primary peritoneal mesothelial cells, murine peritoneal macrophages (WT, *Nlrp3*^*−/−*^, *Casp1*^*−/−*^, *Asc*^*−/−*^)	Frustrated phagocytosis NADPH-dependent ROS production Cathepsin B release K^+^ efflux Increased *NLRP3* and *IL1B* gene expression Involvement of TXNIP Increased Caspase-1 protein levels NLRP3 activation Caspase-1 activation IL-1β secretion IL-18 secretion	Cassel et al. (2008), Dostert et al. (2008), Hillegass et al. (2013), Palomäki et al. (2011), Thompson et al. (2014)
Coal mine dust	BEAS-2B cells, A549 cells, male C57BL/6 mice, male Wistar rats	Production of ROS Increased *NLRP3*, *ASC*, *Caspase 1*, *IL1B* gene expression Increased protein levels of NLRP3 NLRP3 activation Caspase-1 cleavage IL-1β secretion	Zhang et al. (2022a), Zhang et al. (2025)
Other mineral dust particles (stone)	Human bronchial epithelial cells (HBEC3-KT), THP-1 cells	NLRP3 activation Involvement of ASC Caspase-1 activation IL-1β cleavage IL-1β secretion	Grytting et al. (2021)
Titanium dioxide (TiO_2_) nanoparticles	Murine bone marrow-derived macrophages (WT, *Nlrp3*^*−/−*^), murine bone marrow-derived dendritic cells (WT, *Asc*^*−/−*^, *Nlrp3*^*−/−*^), THP-1 cells (WT, *NLRP3*^*−/−*^), Caco-2 cells, HT29 cells, male Wistar rats (oral exposure), female BALB/c mice (inhalation exposure)	Phagocytosis Intracellular accumulation Cathepsin B release Production of ROS Increased *NLRP3*, *Caspase 1*, *IL1B*, *TXNIP* gene expression Involvement of ASC Caspase-1 cleavage IL-1β cleavage IL-1β secretion IL-18 secretion	Abbasi-Oshaghi et al. (2019), Busch et al. (2022a), Duan et al. (2023), Kim et al. (2017), Kolling et al. (2020), Morishige et al. (2010b), Ruiz et al. (2017), Yazdi et al. (2010)
Monosodium urate (MSU) crystals	RAW 264.7 cells, human fibroblast-like synoviocytes, mouse peritoneal macrophages, THP-1 cells	Phagocytosis and cellular internalization Lysosomal rupture Cathepsin B release ROS generation Mitochondrial damage K^+^ efflux Increased *NLRP3*, *ASC*, *Caspase 1* and *IL1B* gene expression Increased protein levels of NLRP3 and Caspase-1 NLRP3 activation Involvement of ASC Caspase-1 activation IL-1β cleavage IL-18 cleavage IL-1β secretion IL-18 secretion	Kim et al. (2020), Martinon et al. (2006), Pan et al. (2021), Pétrilli et al. (2007), Scott et al. (2006), Zheng et al. (2015)
Carbon black	16HBE cells, Sprague–Dawley rats, human corneal epithelial cells, J774A.1 cells, C57BL/6 J mice	Cellular uptake Lysosomal rupture Cathepsin B release Production of ROS Increased *NLRP3*, *Caspase 1*, *ASC* and *IL1B* gene expression Increased NLRP3, Caspase-1, ASC and IL-1β protein levels NLRP3 activation IL-1β release	Long et al. (2020), Wu et al. (2025), Zhou et al. (2020)
Diesel engine exhaust particles	RAW 264.7 cells, C57BL/6 mice (WT, *Nlrp3*^*−/−*^), Balb/c mice	Increased *NLRP3*, *Caspase 1*, *IL1B* and *IL18* gene expression Increased protein levels of NLRP3 and Caspase-1 Involvement of ASC Caspase-1 activation IL-1β secretion IL-18 secretion GSDMD activation Pyroptosis	Li et al. (2020b), Singh et al. (2024), Uh et al. (2017)
Residual oil fly ash	THP-1 cells (*ASC*-GFP reporter), Human peripheral blood mononuclear cells, murine bone marrow-derived macrophages (*Asc*-reporter, *Nlrp3*^*−/−*^, *Casp1*^*−/−*^)	Intracellular accumulation Mitochondrial damage Production of ROS Lysosomal disruption K^+^ efflux Increased *NLRP3*, *Caspase 1* gene expression NLRP3 assembly Involvement of ASC Increased Caspase-1 activity Increased IL-1β protein levels in cell lysates IL-1β secretion IL-18 secretion	Caceres et al. (2024)
Ambient particulate matter (PM2.5)	THP-1 cells (WT, *NLRP3*^−/−^, *ASC*^−/−^), RAW 264.7 cells, murine primary bone marrow-derived macrophages (WT, *Tlr2*^*−/−*^, *Tlr4*^*−/−*^), BEAS-2B cells, Balb/c mice, HUVECs, 16HBE cells, human corneal epithelial cells, murine cornea tissue, ICR mice, C57BL/6 mice, A549 cells	Cellular uptake and internalization TLR2 and TLR4 dependence NFκB activation Lysosomal damage Production of ROS K^+^ efflux Mitochondrial ROS production Increased *NLRP3*, *Caspase-1*, *IL1B* and *IL18* gene expression Increased NLRP3, Caspase-1 and ASC protein levels NLRP3 activation Involvement of ASC Caspase-1 activation IL-1β release IL-18 release GSDMD activation Pyroptosis	Bekki et al. (2016), Cao et al. (2022), He et al. (2019), Jia et al. (2021), Li et al. (2020a), Niu et al. (2021), Shen et al. (2019), Wang et al. (2020), Zheng et al. (2018)
Volcanic ash	Immortalized murine bone marrow-derived macrophages (WT, *Nlrp3*^*−/−*^, *Asc*^*−/−*^, *Casp-1/11*^*−/−*^) primary human peripheral blood mononuclear cells	Cellular uptake Lysosomal disruption Cathepsin B release Production of ROS NLRP3 involvement ASC Involvement Caspase-1 activation IL-1β secretion	Damby et al. (2018)
Desert dust	RAW 264.7 cells, THP-1 cells (WT, *NLRP3*^*−/−*^, *CASP1*^*−/−*^)	Increased *NLRP3*, *ASC* and *IL1B* gene expression Caspase-1 involvement IL-1β secretion	Bredeck et al. (2023a), Bredeck et al. (2023b), He et al. (2010)
Synthetic amorphous silica (SAS), including food grade (E551)	Murine bone marrow-derived dendritic cells (WT, *Nlrp3*^*−/−*^), THP-1 cells (WT, *NLRP3*^*−/−*^), NR8383 cells, HUVECs, BV2 murine microglia cells, RAW 264.7 cells, murine alveolar macrophages (MH-S)	Cellular uptake via phagocytosis NADPH-dependent ROS production Lysosomal rupture Cathepsin B release Increased *IL1B* gene expression Increased protein levels of NLRP3, ASC, Caspase-1, IL-1β NLRP3 activation Involvement of ASC Caspase-1 activation IL-1β cleavage IL-1β secretion GSDMD activation Pyroptosis	Bredeck et al. (2023a), Di Cristo et al. (2015), Hou et al. (2022), Kolling et al. (2020), Liu et al. (2021), Ma et al. (2022), Morishige et al. (2010a), Winkler et al. (2017), Winter et al. (2011)
Carbon nanotubes	Human macrophages, THP-1 cells (WT/*NLRP3*^*−/−*^, *ASC*^*−/−*^, *P22*^*phox−/−*^), C57BL/6 mice (WT, *P47*^*phox−/−*^), murine primary bone marrow-derived macrophages (WT, *P47*^*phox−/−*^), primary human monocytes	Phagocytosis NADPH-dependent ROS production Lysosomal rupture Cathepsin B release K^+^ efflux Mitochondrial ROS production Tyrosine kinase activation NLRP3 activation Involvement of ASC Caspase-1 activation IL-1β secretion GSDMD activation Pyroptosis	Busch et al. (2022a), Cui et al. (2014), Lim et al. (2025), Meunier et al. (2012), Palomäki et al. (2011), Sun et al. (2015), Wang et al. (2012)
Silver nanoparticles	HepG2 cells, THP-1 cells, primary human macrophages, PBMC-derived monocytes	Cellular uptake and internalization Lysosomal dysfunction Cathepsin B release K^+^ efflux ROS generation Mitochondrial damage NLRP3 activation Caspase-1 cleavage Increased Caspase-1 activity IL-1β secretion Pyroptosis	Mishra et al. (2016), Simard et al. (2015), Yang et al. (2012)
Cobalt nanoparticles	Normal human liver L02 cells, C57BL/6 J mice, BV2 cells	Intracellular uptake Intracellular ROS Increased NLRP3, Caspase-1 and IL-1β protein levels NLRP3 activation Caspase-1 activation IL-1β secretion IL-18 secretion	Feng et al. (2020), Li et al. (2021)
Amino-functionalized polystyrene (PS-NH_2_) nanoparticles	Human macrophages, THP-1 cells (WT, *NLRP3*^*−/−*^, *CASP1*^*−/−*^), human brain astrocytoma 1321N1 cells	Cellular uptake via phagocytosis and macropinocytosis Lysosomal uptake Lysosomal rupture Cathepsin B release Mitochondrial ROS production TXNIP dissociation NLRP3 assembly Caspase-1 activation IL-1β secretion	Busch et al. (2022a), Busch et al. (2022b), Lunov et al. (2011a), Lunov et al. (2011b), Wang et al. (2013)
Alum	PBMC-derived human dendritic cells, human PBMCs, Bone marrow-derived murine mononuclear cells, THP-1 cells	Lysosomal destabilization Cathepsin B dependence NLRP3 activation Involvement of ASC Caspase-1 activation IL-1β secretion IL-18 secretion	Hornung et al. (2008), Li et al. (2007), Li et al. (2008)
Calcium phosphate crystals such as hydroxyapatite or calcium pyrophosphate	Primary murine macrophages (C57BL/6 WT, *Nlrp3*^*−/−*^, *Casp1*^*−/−*^, *Asc*^*−/−*^), CT26 cells, primary cultured mouse dendritic cells, THP-1 cells, murine bone marrow-derived macrophages (WT, *Nlrp3*^*−/−*^), C57BL/6 mice (WT, *Casp1*^*−/−*^, *Asc*^*−/−*^, *IL-1R*^*−/−*^)	Cellular uptake and internalization Lysosomal uptake Mitochondrial ROS production K^+^ efflux Increased protein levels of NLRP3, Caspase-1, GSDMD, NF-κB, pro-IL-18, pro-IL-1β NLRP3 activation Involvement of ASC Caspase-1 cleavage Caspase-1 activation IL-1β cleavage IL-18 cleavage IL-1β secretion IL-18 secretion GSDMD cleavage Pyroptosis	Campillo-Gimenez et al. (2018), Chen et al. (2024a), Jin et al. (2011), Lebre et al. (2017), Martinon et al. (2006), Maruyama et al. (2021), Xia et al. (2022), Zhang et al. (2022b)
Cholesterol crystals	Human PBMCs, primary human monocytes, primary human monocyte-derived macrophages, primary murine alveolar macrophages (WT and *Nlrp3*^−/−^) THP-1 cells, murine macrophages (WT, *Nlpr3*^−/−^, *Asc*^−/−^), C57BL/6 mice, *Ldlr*^*−/−*^ C57BL/6 mice, human carotid plaques, human decidual biopsy tissues	Cellular uptake and internalization Lysosomal dysfunction Cathepsin B release K^+^ efflux Increased *NLRP3* and *IL1B* gene expression Involvement of ASC ASC speck formation Caspase-1 activation IL-1β cleavage IL-1β secretion IL-18 release GSDMD activation	Duewell et al. (2010), Giambelluca et al. (2025), Niyonzima et al. (2020), Rajamäki et al. (2010), Samstad et al. (2014), Silva et al. (2020), Yalcinkaya and Tall (2025)
Cerium oxide nanorods (high aspect ratio)	THP-1 cells (WT, *NLRP3*^*−/−*^, *ASC*^*−/−*^)	Lysosomal rupture Cathepsin B dependence NLRP3 activation Involvement of ASC IL-1β secretion	Ji et al. (2012b)
Copper oxide nanoparticles	J774A.1 cells	Lysosomal damage Cathepsin B release ROS generation Increased *NLRP3*, *Caspase 1* and *IL1B* gene expression NLRP3 activation Caspase-1 activation IL-1β secretion	Tao et al. (2021)
Cromium oxide nanoparticles	Murine bone marrow-derived macrophages (WT, *Nlrp3*^*−/−*^, *Casp1*^*−/−*^)	Lysosomal damage Cathepsin B-dependence IL-1β secretion	Abdoulkader et al. (2025)
Graphene oxide nanomaterials	Human PBMC-derived macrophages	Cellular uptake and internalization Lysosomal rupture K^+^ efflux NADPH-dependent intracellular ROS production Cathepsin B release Involvement of ASC Caspase-1 activation IL-1β secretion	Mukherjee et al. (2018)
Iron oxide nanoparticles	Murine bone marrow-derived dendritic cells, human whole blood, RAW264.7 cells, murine bone marrow-derived macrophages (WT, *Nlrp3*^*−/−*^, *Asc*^*−/−*^, *Casp1*^*−/−*^)	Intracellular uptake Intracellular ROS Lysosomal damage K^+^ efflux Increased protein levels of Caspase-1 and pro-IL-1β NLRP3 activation Involvement of ASC Caspase-1 activation IL-1β secretion GSDMD activation Pyroptosis	de Melo et al. (2022), Liu et al. (2018), Stroh et al. (2004), Wolf-Grosse et al. (2017)
Nickel oxide nanoparticles	RAW264.7 cells, Sprague Dawley rats, C57BL/6 J mice (WT, *Nlrp3*^*−/−*^), MH-S cells	Phagocytosis ROS generation Increased *NLRP3*, *IL1B* and *IL18* gene expression Increased protein levels of NLRP3 and Caspase-1 NLRP3 activation Caspase-1 activation IL-1β secretion IL-18 secretion	Cao et al. (2016), Mo et al. (2025)
Cadmium telluride quantum dots	ICR mice, KUP5 cells	Intracellular uptake Intracellular ROS Increased protein levels of NLRP3 and Caspase-1 Caspase-1 activation IL-1β secretion	Pang et al. (2021)
Cellulose nanocrystals	J774A.1 cells, PBMCs, THP-1, murine BMDCs	Cellular Uptake Lysosomal damage Cathepsin B release Intracellular ROS Mitochondrial ROS production Mitochondrial damage Increased protein levels of NLRP3 and pro-IL-1β NLRP3 assembly Caspase-1 activation IL-1β secretion	Guglielmo et al. (2017), Sunasee et al. (2015), Wang et al. (2019)

## NLRP3 activation mechanisms triggered by particles and fibers

**Fig. 1:**
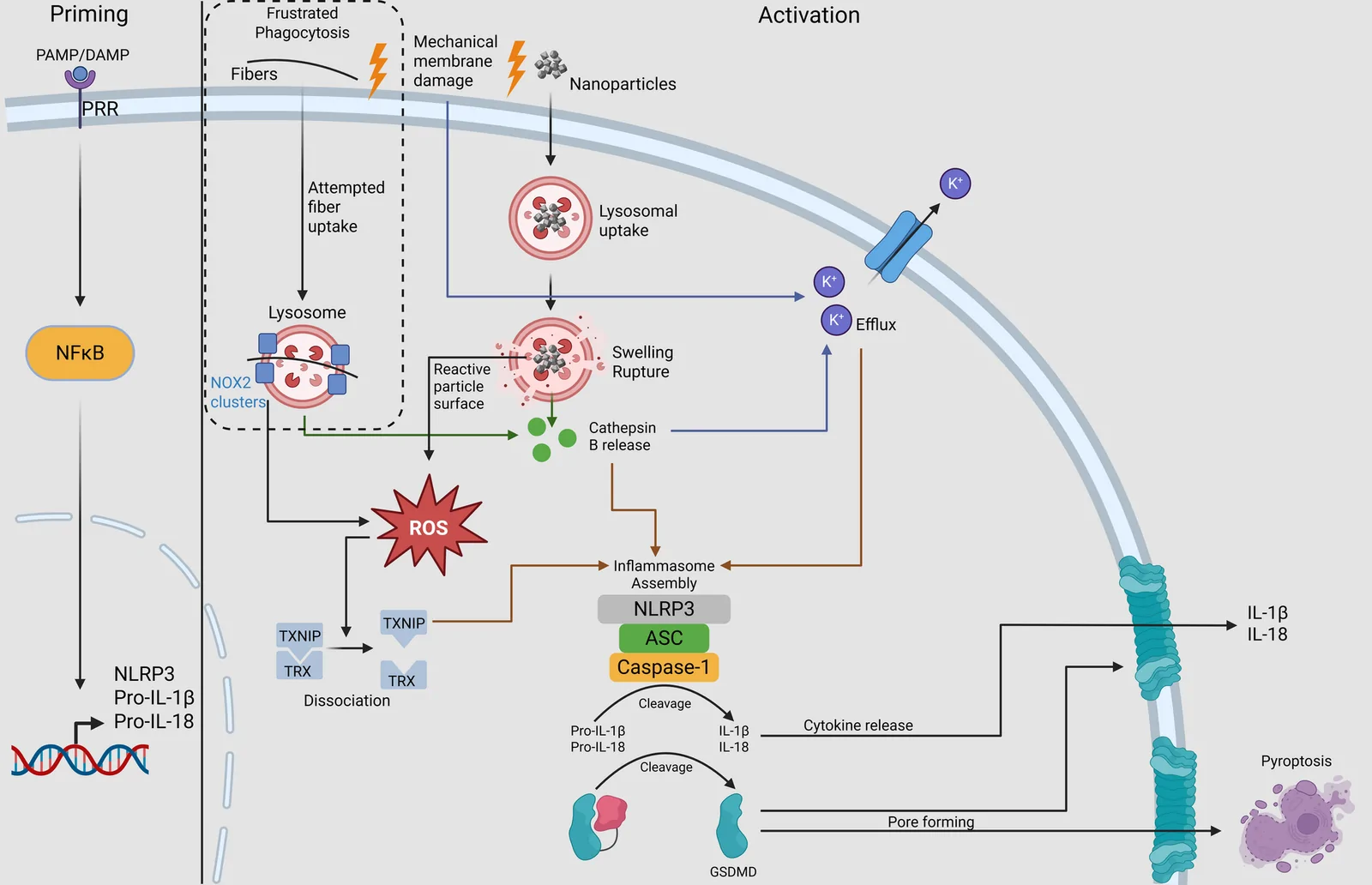
NLRP3 activation mechanisms triggered by particles and fibers. Binding of a pathogen- or danger-associated molecular pattern (PAMP/DAMP) to a pattern recognition receptor (PRR) induces priming of the NLRP3 inflammasome via NF-κB-mediated transcription of *NLRP3*, *IL1B* and *IL18*. In case of fibers, unsuccessful engulfment, so-called frustrated phagocytosis, leads to mechanical damage in the plasma membrane of both cell and lysosome, activating NOX2 clusters to generate intracellular reactive oxygen species (ROS). Successful engulfment of small particles in lysosomes leads to swelling and finally rupture of lysosomes, releasing Cathepsin B into the cytosol. The reactive surface of particles released from the ruptured lysosome generates intracellular ROS as well. The mechanical damage caused by fibers and sharp particles to the cell membrane causes efflux of potassium ions (K^+^). The release of Cathepsin B into the cytosol is suspected to also cause K^+^ efflux, but the mechanism is poorly understood. The dissociation of TXNIP from TRX due to ROS scavenging, the release of Cathepsin B, and K^+^ efflux are capable of triggering the assembly and activation of the NLRP3 inflammasome complex, which includes activation of Caspase-1, which can convert pro-IL-1β and pro-IL-18 into their mature forms via cleavage. Caspase-1 also cleaves Gasdermin D (GSDMD), which is capable of forming pores in the cell membrane, allowing IL-1β and IL-18 to be released in the extracellular environment, but ultimately leads to cell death via pyroptosis. Created with BioRender.com

The NLRP3 inflammasome can be activated by a variety of different stimuli, such as bacterial toxins, ROS or cellular stress, detected by surface receptors or intracellular sensor proteins. In the case of particles, several dominant events lead to the activation of the NLRP3 inflammasome (Fig. 1). For most particles, the first event in this cascade is the uptake and internalization by cells, typically specialized immune cells such as macrophages, neutrophils or dendritic cells (Uribe-Querol and Rosales 2020). Specific uptake mechanisms, e.g. phagocytosis, micropinocytosis or a type of endocytosis might be preferred, depending on the particle size (Unfried et al. 2009). Particles taken up via these pathways end up in lysosomes, cellular structures serving the purpose of digesting and destroying endocytosed pathogens or other foreign matter via acidification, enzymatic degradation and ROS generation (Luzio et al. 2007). Specific particle properties, such as sharp edges due to crystallinity, or high surface reactivity, can rupture lysosomes, leading to the release of lysosomal enzymes into the cytosol, which can activate the NLRP3 inflammasome (Bai et al. 2018). This link is well established, as lysosomal rupture alone, without the presence of particles, is already sufficient to activate the NLRP3 inflammasome (Hornung et al. 2008). Of note, specific types of engineered nanomaterials, such as graphene, seem to be able to activate NLRP3 via TLRs without the need for cellular uptake and internalization (Qu et al. 2013).

In the case of particles with an amine-modified surface, which have been shown to be potent NLRP3 activators, another potential mechanism, the so-called “proton sponge”, has been hypothesized. Originally, the proton sponge hypothesis was discussed to explain the observed phenomenon of cationic drug carriers escaping lysosomes (Vermeulen et al. 2018). After lysosomal uptake, amine-modified particles might stall acidification of the lysosomes by absorbing protons via the amine group (-NH_2_ to -NH_3_^+^), leading to more protons being pumped into the lysosomes, causing counterions and water to follow (Behr 1997). This could ultimately lead to swelling and rupture of the lysosomes, activating the NLRP3 inflammasome via Cathepsin B release. Although still heavily debated, the proton sponge hypothesis might at least partly explain the strong NLRP3 activating potential of amine-modified polystyrene particles (Busch et al. 2022a), for which lysosomal rupture has been reported previously (Lunov et al. 2011b), despite not having a crystalline structure.

Another mechanism of NLRP3 activation by specific types of particles is the generation of ROS on their surface. Predominantly metal and metal-oxides, but also silica-based particles that function as NLRP3 activators, have been shown to generate ROS via their reactive surface (Hellack et al. 2017; Lehman et al. 2016). For example, quartz particles harbor free radicals on their surface due to homolytic cleavage of silicon-oxygen bonds during fracturing, and are able to generate H_2_O_2_, HO· and other ROS in aqueous suspensions (Fubini et al. 1990; Konecny et al. 2001). Nanoparticles composed of, or containing, transition metals (e.g. iron, copper, cobalt, etc.) on their surface can generate ROS via Fenton and Fenton-like reactions (Leonard et al. 1998; Sicwetsha et al. 2021; Voinov et al. 2011). In the case of coal mine dust, the iron component of coal has been suggested to be the dominant active agent that causes lung inflammation via ROS generation, in contrast to the coal dust particle itself (McCunney et al. 2009). Similarly, the iron content in asbestos has been linked to ROS generation (Kamp et al. 1992; Weitzman and Graceffa 1984), contributing to the toxicity of asbestos fibers. Interestingly, in the case of Libby amphibole, a mixture of mineral fibers similar to asbestos, additional iron loading is associated with a dampened inflammasome response (Shannahan et al. 2012), presumably via altered fiber reactivity and signaling inhibition upstream of NLRP3.

Generation of ROS via an NADPH oxidase (NOX2)-dependent respiratory burst is a dominant mechanism for fibers, such as asbestos and carbon nanotubes (Dostert et al. 2008; Elgrabli et al. 2015). The physiological function of NOX2 is superoxide production to destroy microbial pathogens in the phagosomes (Noreng et al. 2022). It appears that particle-associated NOX2 activation is fiber shape-specific, as it was shown that NOX2 clusters at the plasma membrane increase in numbers during frustrated phagocytosis of carbon nanotubes (Joly et al. 2020), while NOX2 activation was not observed for silver nanoparticles, carbon black or TiO_2_, despite phagolysosomal localization (Sun et al. 2015) and being well-established NLRP3 activators. The fiber-induced prolonged NOX2 activation generates intracellular ROS that triggers activation of the NLRP3 inflammasome. The ability of fibers to trigger NLRP3 activation via this pathway heavily depends on their rigidity, length, and aspect ratio (Wang et al. 2017).

On their surface, particles can carry PAMPs that contribute to the priming or the activation of the NLRP3 inflammasome. Ambient PM2.5 and desert dust have been found to contain bacterial LPS and β-glucan (Bekki et al. 2016; He et al. 2019). After heat-inactivating these PAMPs, the capacity to trigger NLRP3-dependent IL-1β secretion was substantially decreased. Additionally, the endotoxin neutralizer polymyxin B was shown to attenuate IL-1β secretion, and TLR2 and TLR4 contributed to mediating the IL-1β secretion in response to ambient PM2.5 (Bekki et al. 2016).

In summary, the ability to rupture lysosomes, ROS-generating potential of the surface, fiber-like properties, and absorbed PAMPs seem to be the main drivers behind NLRP3 priming and activation via particles.

## Human evidence

There is only limited direct evidence of NLRP3 activation by particles in humans, and most of the available data is derived from epidemiological studies in occupational settings. Although the hazards of several particles towards human health are well established, there is only a limited number of studies pointing towards an involvement of NLRP3 in pathogenesis, primarily through measurement of IL-1β levels in blood. Workers in Swedish iron foundries, exposed to high concentrations of respirable dust, showed IL-1β/IL-18 plasma levels and Caspase-1 activation correlating with exposure levels (Hedbrant et al. 2020, 2023). Workers exposed to nanoscale carbon black had significantly increased serum levels of pro-inflammatory cytokines, including IL-1β, compared to the control group, suggesting involvement of NLRP3 in this adverse reaction towards the inhaled particles (Zhang et al. 2014). Occupational exposure to multi-walled carbon nanotubes in a large-scale manufacturing company in Russia was associated with significantly increased IL-1β concentrations in serum and sputum of workers (Fatkhutdinova et al. 2016). Coal miners in Turkey suffering from pneumoconiosis or progressive massive fibrosis showed increased IL-1β concentrations in serum and bronchoalveolar lavage samples (Ulker et al. 2008). Further, alveolar macrophages harvested from coal workers’ pneumoconiosis patients showed higher levels of spontaneous IL-1 secretion than healthy controls (Lassalle et al. 1990). These findings correlate well with the NLRP3-activating effects of particles observed in respective in vitro studies (Table 1).

Other studies show correlations between a specific NLRP3 polymorphism and disease onset, for example, certain NLRP3 polymorphisms in workers occupationally exposed to asbestos fibers were associated with a decreased (Franko et al. 2020) or increased (Kukkonen et al. 2014) risk of developing asbestosis, depending on the polymorphism. In addition, an increased risk of pneumoconiosis was observed in coal workers in a Chinese population bearing a specific NLRP3 polymorphism (Ji et al. 2012a). However, the authors do not indicate which influence this polymorphism could have on NLRP3 activity, while most likely, a polymorphism increasing the activity of NLRP3 would lead to a higher chance of developing a disease.

## Immunological diseases linked to particle exposure via the NLRP3 inflammasome pathway

Being a mechanism of the innate immune system, inflammation plays an important role in the defense against invading pathogens. However, persistent particle-induced inflammation can lead to the development of immunological diseases. This has been mainly described for the lung, as diseases such as asbestosis, silicosis and coal workers’ pneumoconiosis are directly linked to the respective occupational exposures. Long-term exposure and accumulation of inhaled particles causes persistent inflammatory reactions in the lung, which eventually lead to fibrosis (Savin et al. 2022). The progressing fibrogenesis in lung tissue impairs gas exchange and makes breathing difficult (Plantier et al. 2018). Furthermore, lung diseases such as pneumoconiosis have been shown to be a risk factor for lung cancer (Hubbard et al. 2000).

Several of these lung diseases that are linked to particle exposure have been shown to be associated with activation of the NLRP3 inflammasome in animal models (Table 2). In these studies, involvement or even requirement of NLRP3 is usually shown either via correlating NLRP3-specific markers with the disease scoring, or by preventing or ameliorating disease onset or progression via NLRP3 inhibition, knockdown or knockout. Early rodent studies showed decreased inflammatory parameters in *Nlrp3*^*−/−*^ mice after intranasal instillation exposure to crystalline silica (Cassel et al. 2008) or 9-day inhalation of asbestos (Dostert et al. 2008), establishing a clear role of NLRP3 in the pathogenesis of silicosis and asbestosis. Other studies showed that exposure of Wistar rats to a single dose of crystalline silica via instillation induced time-dependent hallmarks of silicosis that correlated well with Caspase-1 and Il-1β immunohistochemistry scoring (Peeters et al. 2014). *Nlrp3*^*−/−*^ and *Casp1*^*−/−*^ mice showed reduced thickening of peritoneal walls and peritoneal submesothelium after asbestos exposure compared to wildtype animals (Thompson et al. 2017). Thickening of peritoneal walls and the submesothelium is a consequence of scar tissue formation, typical after asbestos exposure. Carbon black nanoparticles induced pulmonary fibrosis in Sprague–Dawley rats, which could be partially rescued by NLRP3 inhibition via Forkhead transcription factor class O (FOXO)3a suppression (Zhou et al. 2020), a transcription factor that was recently found to promote *Nlrp3* transcription, similar to NF-κB (Shen et al. 2021). Furthermore, the NLRP3 inflammasome has been demonstrated to be essential for the development of COPD in C57BL/6 mice (Yang et al. 2015). Interestingly, PM2.5 induced hallmarks of COPD in ICR mice via NLRP3-associated mechanisms such as IL-1β and IL18 expression, suggesting that NLRP3 activation via PM2.5 contributes to the development of COPD (Li et al. 2020a). A plausible link between PM2.5 and COPD has already been supported in much earlier epidemiological studies (Schikowski et al. 2010, 2005). Further, production of transforming growth factor (TGF)-β, an important molecular mediator of fibrosis (Fernandez and Eickelberg 2012), after inhalation exposure to PM2.5 in mice, correlates well with IL-1β release in macrophages, suggesting a link between NLRP3 activation and a PM2.5-mediated progression of lung fibrosis (Cao et al. 2022). This is also supported by much earlier studies showing a link between NLRP3 activation and lung fibrosis in the case of crystalline silica (Dostert et al. 2008).

**Table 2:** Diseases linked to particles via NLRP3 inflammasome activation

Particle type	Disease	Models	Observed events	References
Crystalline Silica	Silicosis	Female Wistar rats, C57BL/6 mice (WT, *Nlrp3*^*−/−*^, *Asc*^*−/−*^, *Nlrp3*-CHCl reporter)	Silicotic nodules Granulomas Deposition of collagen Immune cell infiltration	Cassel et al. (2008), Lam et al. (2025), Peeters et al. (2014)
PM2.5	COPD	ICR mice/COPD mouse model	Thickened alveolar walls Neutrophil infiltration	Li et al. (2020a)
Asbestos fibers	Asbestosis/Malignant mesothelioma	C57BL/6 mice (WT, *Nlrp3*^*−/−*^, *Casp1*^*−/−*^, *Asc*^*−/−*^)	Mesothelial to fibroblastic transition Immune cell infiltration Thickened peritoneal walls	Chow et al. (2012), Dostert et al. (2008), Thompson et al. (2017)
Coal mine dust	Coal workers’ pneumoconiosis/pulmonary fibrosis	C57BL/6 mice, Wistar rats	Epithelial-mesenchymal transition Increased fibrosis scores	Zhang et al. (2022a), Zhang et al. (2025)
Carbon black	Pulmonary fibrosis	Sprague–Dawley rats	Immune cell infiltration Thickened alveolar walls	Zhou et al. (2020)
Carbon Nanotubes	Pulmonary fibrosis	C57BL/6 mice	Increased collagen content in lung tissue	Sun et al. (2015), Wang et al. (2012)
Food grade TiO2 (E171)	Colitis	DSS-induced colitis C57BL/6 mice (WT, *Nlrp3*^*−/−*^)	Colon shortening Mucosal damage Increased infiltration and histological scores	Ruiz et al. (2017)
MSU crystals	Gout	C57BL/6 J mice (WT/ *Nlrp3*^*−/−*^, *Casp1*^*−/−*^, *Asc*^*−/−*^, *IL-1β*^*−/−*^, *IL-1RI*^*−/−*^)	Neutrophil infiltration Hypernociception	Amaral et al. (2012)

An animal study focusing on intestinal effects of food grade TiO_2_ particles (E171) found a worsening of pre-existing colitis, a type of inflammatory bowel disease located in the colon, which was not observed in *Nlrp3* knockout mice (Ruiz et al. 2017). In healthy animals, however, chronic exposure to TiO_2_ did not result in increased IL-1β or IL-18 levels, suggesting that NLRP3 was not directly activated in healthy mice (Bettini et al. 2017). An increase in susceptibility towards potential toxicants due to pre-existing intestinal inflammation appears to be a common phenomenon in toxicology (Walraven et al. 2024).

The first disease that was found to be related to particles via NLRP3 activation is gout, a painful form of arthritis. Although it is not related to exogenous particle exposure, it is one of the best studied examples of NLRP3 activation via particles, uncovering the pivotal role of the inflammasome in the pathogenesis of a disease for the first time (Dagenais et al. 2012; Martinon et al. 2006). In gout, excess or insufficient excretion of urate leads to deposition in the form of monosodium urate (MSU) crystals in peripheral joints, typically of the big toe (Emmerson 1996). These MSU crystals cause joint inflammation and pain. In a murine gout model, MSU crystals were injected into the knee joint, leading to pain and inflammation via neutrophil infiltration (Amaral et al. 2012). In *Nlrp3*^*−/−*^, *Asc*^*−/−*^, and *Casp1*^*−/−*^ mice, all inflammatory parameters and nociception were significantly reduced compared to the WT animals, pointing towards a crucial role of NLRP3 in gout onset and progression.

Other endogenous particles are known to cause diseases via the NLRP3 inflammasome pathway as well, but are beyond the scope of this work. This includes Alzheimer’s disease, in which amyloid protein aggregates activate NLRP3 similar to particles (Van Zeller et al. 2021), atherosclerosis, linked to NLRP3 activation via cholesterol crystals (Yalcinkaya and Tall 2025), and kidney disease due to renal formation of MSU crystals (Wang et al. 2022).

## NLRP3 activation as a biosensor tool for particle hazard assessment

Based on the well-studied connections between particle-induced NLRP3 activation and adverse health outcomes, including the potential development of diseases, NLRP3 activation provides a mechanistically anchored readout for particle hazard assessment. Accordingly, it can be applied to early-stage material screening, regulatory hazard identification, and ranking within material classes. Previous studies have used simple in vitro screening tools employing immune cells, such as bone marrow-derived macrophages (BMDMs), NR8383 or THP-1 cells, in order to rank panels of nanomaterials by hazard, based on their potency to activate NLRP3 derived from the magnitude of IL-1β secretion (Busch et al. 2022a; Kolling et al. 2020). However, interpretation is strongest when NLRP3 readouts are integrated with particle characteristics that determine in vivo persistence and outcome, rather than used in isolation (Wang et al. 2017). In this context, biopersistence, mainly connected to solubility, is often a key discriminator, because transient in vitro signals from rapidly dissolving materials may not translate to sustained lung burden, whereas poorly soluble and biopersistent materials are more likely to drive chronic inflammation and fibrosis (Cassel et al. 2008; Wang et al. 2017).

Although IL-1β secretion in vitro is a useful qualitative measure of NLRP3 activation, the magnitude of IL-1β secretion does not always correlate with the intrinsic hazard of the particle type when comparing different studies. While the applied model and exposure strategy needs to be chosen carefully, the effect level of IL-1β alone only allows a hazard ranking within the same in vitro model. In a very simplistic approach, we have previously compared IL-1β secretion in THP-1-derived macrophages to the outcome of a mouse study, after exposure to two different types of carbon nanotubes. In both studies, the longer, more rigid nanotubes showed stronger pro-inflammatory effects (Busch et al. 2022a; van Berlo et al. 2014).

Besides the NLRP3 inflammasome, other inflammasomes such as NLRP1, NLRC4 and AIM2 can also induce the activation of caspase-1 and the secretion of IL-1β (Kaushal et al. 2015; Sasaki et al. 2020; Zhang et al. 2016). However, these inflammasomes are mainly involved in responses associated with pathogens and have, to the best of our knowledge, not been observed to be activated by particles. Nevertheless, biologically active components (viral/bacterial) located on the particle surface might cause inflammasome activation that is not directly related to an actual particle-related mechanism (Bredeck et al. 2023a), for example through interaction with PRRs. Yet, an overwhelming amount of data shows that in the case of particle exposure, IL-1β secretion is a reliable biomarker for NLRP3 activation (see Table 1), especially when used in an experimental setup that includes NLRP3 knockouts or inhibitors as mechanistic proof.

For practical screening, it is advisable to include a well-characterized reference particle and verify NLRP3-dependence so that IL-1β and IL-18 signals are not misassigned to non-inflammasome effects. A useful approach is to include a class-matched benchmark particle control that confirms particle handling and a relevant upstream pathway. For particle-type materials, either MSU crystals or a crystalline silica reference can be included as reference, whereas for high-aspect-ratio materials, a long, rigid carbon nanotube can serve as an asbestos-like reference for frustrated phagocytosis-type pathways. In addition, a non-particulate NLRP3 activator (e.g. ATP or nigericin) can be included as a pathway competence positive control, as they are less sensitive to dispersion or delivery artifacts (Vandebriel et al. 2022).

Considering the delivered dose in vitro is essential when comparing materials, because comparative ranking based on IL-1β and IL-18 secretion is not defensible unless the delivered dose is accounted for (OECD 2025). This is particularly relevant when agglomeration, sedimentation dynamics, or effective density differ between the materials (DeLoid et al. 2014). Therefore, it is advisable to report at least an area-normalized dose (µg cm^−2^) and, where feasible, a delivered dose estimate, complemented by surface area or particle number, when informative for the material class.

From a practical perspective, it is further recommendable to test dose-dependency and to interpret inflammasome readouts together with parallel cytotoxicity and assay-interference checks. The highest concentration is ideally chosen as the top dose that avoids nonspecific lysis while maintaining interpretable readouts, bearing in mind that certain types of cell death, such as pyroptosis, can be mechanistically linked to inflammasome activation (Cullen et al. 2015). Because microbial ligands can dominate priming, it is advisable to control for endotoxin or other PAMP contaminations (Li et al. 2017; Smulders et al. 2012).

## NLRP3 activating potential of emerging contaminants, novel materials and unregulated natural particles

With increasing environmental pollution on the one hand, and the continuously developing field of nanotechnology on the other hand, there is a large panel of emerging anthropogenic sources of particles that might result in human exposure. These sources include environmental micro- and nanoplastic particles that end up in food and drinking water, novel engineered nanomaterials (ENMs) that are used as food additives or coatings of furniture and textiles, and particulate drug-carriers in the field of nanomedicine. However, there are also still under-studied natural particles with incomplete human risk assessment or lacking defined regulatory frameworks, such as desert dust (Bredeck et al. 2024) or particles derived from wildfires (Krupnick et al. 2025; Orr et al. 2025).

Micro- and nanoplastic particles are released from plastic waste in the environment via embrittlement and abrasion and have contaminated a significant fraction of foodstuffs and drinking water (Cox et al. 2019). Due to the large variety of different polymers, shapes and sizes, micro- and nanoplastics simply represents an umbrella term for a large number or different types of particles. Previously, we investigated the NLRP3 activating potential of selected types of micro- and nanoplastics in macrophage-like cells in vitro, but did not find any evidence of NLRP3 activation (Busch et al. 2022a). Amino-modified polystyrene particles were the exception, which already showed a strong IL-1β release and clear hallmarks of NLRP3 activation in another study (Lunov et al. 2011b). However, amine-modified polystyrene is highly unlikely to form from plastic waste in the environment and is therefore not an actual representative of micro- and nanoplastics. Interestingly, a number of studies report NLRP3 activation by polystyrene particles in animal studies (Chi et al. 2022; Hou et al. 2021; Mu et al. 2022; Wei et al. 2021), mainly at unrealistically high exposure concentrations (Alijagic et al. 2023), which is not reflected in comparable in vitro studies. These high concentrations might cause systemic NLRP3 activation in animals not via particle-intrinsic potential, but via side effects like mechanical damage of the GI-tract leading to systemic inflammation (Chen et al. 2024b).

Novel ENMs, sometimes called advanced materials, for example core–shell materials or nanocomposites, are attracting more attention due to their potential to improve the materials properties, combine the properties of multiple materials in a single particle, or possess completely new properties that are beneficial for the intended applications, e.g. industrial manufacturing, antimicrobial potential in food packaging, or biomedicine (Cheng et al. 2025; Ghosh Chaudhuri and Paria 2012; Ndayishimiye et al. 2022). With an astonishing number of ENM patents filed every year, the nanotechnology industry and related markets are expected to grow rapidly in the future (Sohail 2025). In this context, the safe-and-sustainable-by-design concept (SSbD) is gaining popularity (Chakraborty et al. 2025), including toxicity testing in the process of material development, e.g. novel insulation materials (Di Battista et al. 2024). This concept can also be applied to novel plastic products with concurrent testing of true-to-life micro- and nanoplastic particles (Brouwer et al. 2025; Brunelli et al. 2025). The idea of using nanoparticles for targeted drug delivery through exploitation of the particles’ unique pharmacokinetics has already resulted in a large number of FDA-approved nanocarriers, including polymeric, crystalline and metal-based particles (Yusuf et al. 2023). As established by Wang et al. (2017), there are several key structure activity relationships (SARs) determining the NLRP3 activating potential and therefore hazardousness of ENMs, e.g. surface reactivity of carbon nanotubes, high aspect ratios of nanorods or -wires, biotransformation of rare earth-oxide ENMs, or the density of silanol groups on the surface of silica-based ENMs (Wang et al. 2017). Knowledge of these SARs is crucial when aiming for a safer design of novel ENMs. Nevertheless, large-scale production or application of novel ENMs in patients and consumer products will still require a toxicological hazard assessment in both occupational safety and consumer safety contexts, especially if these novel materials are based on known NLRP3 activators, such as TiO_2_ or SiO_2_.

A good example of a class of novel ENMs are cellulose nanocrystals, a fiber-shaped material characterized by versatile applicability in many industrial branches (Aziz et al. 2025). Gaining popularity in the early 2010s, some types of cellulose nanocrystals have been shown to be able to activate the NLRP3 inflammasome in vitro (Guglielmo et al. 2017; Sunasee et al. 2015; Wang et al. 2019), while causing inflammation (including IL-1β secretion) and early signs of lung fibrosis after inhalation in vivo (Shvedova et al. 2016), although generally less pronounced than asbestos or MWCNTs (Ede et al. 2019).

Regarding unregulated natural particles, we previously assessed IL-1β release in THP-1 cells after exposure to PM2.5 and PM10 samples collected during Saharan dust events, compared to samples from a reference period (Bredeck et al. 2024). Current air quality regulations do not require authorities to take action if PM thresholds are exceeded because of desert dust events (EPA 1971; European Commission 2022). Particles collected during the dust events showed higher intrinsic inflammatory potency than particles collected during the reference period, indicating that transported desert dust can potentiate the inflammatory potential of local PM. This is in line with epidemiological studies reporting increased respiratory morbidity and mortality from desert dust exposure (Kashima et al. 2016; Mallone et al. 2011; Stafoggia et al. 2016).

## Summary and conclusions

Especially for work-related exposure to particles, clear connections between activation of the NLRP3 inflammasome and the development of diseases could be shown on several levels, ranging from in vitro experiments reporting IL-1β release upon particle exposure, to extensive in vivo studies showing immune cell infiltration and lung inflammation and fibrosis. Based on this well-established link between NRLP3 activation via particles and the development of diseases, we encourage testing for NLRP3 activation in simple in vitro models as a first step in assessing the potential hazard of new particulate materials such as nanocomposites or core–shell nanoparticles, as well as emerging environmental particulate pollutants such as micro- and nanoplastics. In addition, we encourage expanding ambient PM screening strategies (PM2.5, PM1, ultrafine particles) and interpreting these data in combination with epidemiological studies.

## Data Availability

No datasets were generated or analysed during the current study.
